# The neuronal membrane as a key factor in neurodegeneration

**DOI:** 10.3389/fphys.2013.00188

**Published:** 2013-07-18

**Authors:** Raquel Marin

**Affiliations:** Laboratory of Cellular Neurobiology, Department of Physiology, School of Medicine, La Laguna UniversityLa Laguna, Spain

The neuronal membrane is the site where most processes involved in neuronal preservation and functioning are triggered. These actions require the participation of membrane-related molecular agents, which associate in protein/lipid clusters to initiate molecular processing and signal transduction. Furthermore, numerous neuropathological phenomena have their origin in this structure and/or are a consequence of membrane dysfunctioning (Lukiw, 2013). Even though the precise molecular mechanisms to preserve “membrane health” are unclear in most cases, it seems that the effects of different lipids on the microstructural and stoichiometric properties of the membrane influence the behavior and association of proteins within the lipid environment, which determine the final cell response.

This Special issue composed by five outstanding Reviews and a very interesting Hypothesis and Theory work tackles different strategies and events taking place at the neuronal membrane related to neurodegeneration and neuroprotection against the most common form of dementia in the elderly, Alzheimer's disease (AD) and Parkinson's disease (PD). These diseases are characterized by pathological protein aggregates in particular brain areas leading to selective neurodegeneration that explains their clinical phenotypes. Related to AD, one of the main neuropathological hallmarks courses with amyloid beta [Aβ peptide formation, produced by amyloid precursor protein (APP) proteolysis, through the action of two secretases: β-secretase (BACE) and γ-secretase]. In particular, BACE1 is the main responsible for Aβ generation, and is therefore considered a major drug target for AD. However, as reviewed in Dislich and Lichtenthaler (2012), BACE1 also catalyzes the proteolysis of other different substrates still not well-characterized, and whose identification is crucial to develop better pharmacological strategies targeting this molecule. Another important aspect awaiting clarification is related to the involvement of membrane lipids in BACE1 regulation. Thus, this enzyme can be palmitoylated, and anchored into lipid raft microstructures by GPI binding, also observing an important influence on BACE1 activity and Aβ production depending on cholesterol levels (Dislich and Lichtenthaler, 2012).

Thus, cholesterol is crucial to keep the neuronal membrane architecture stable, and failures in cholesterol homeostasis contribute to progressive neurodegeneration (reviewed in Anchisi et al., 2013). Cholesterol is an essential component of myelin, and it is thereby required for brain growth, differentiation, and preservation. This sterol also participates in the particular composition of membrane raft microdomains, which are main sites of protein signaling clusters related to neuroprotection (reviewed in Marin, 2011). Even though cholesterol is mainly found free in the brain, the small proportion (~5%) of esterified cholesterol is modulated by brain growth and increase myelination, evoking the exquisite regulation of this sterol. Indeed, cholesterol impairment in the neuronal membrane has been related, among others, to AD, prion diseases and autistic spectrum disorders, as it has been nicely illustrated by Anchisi et al. (2013). Furthermore, an important factor of cholesterol production and trafficking is due to the activity of low density lipoprotein receptor-related proteins (LRP1 and LRP2) which regulate cholesterol uptake into the neuron. Even though LRPs can bind to numerous extracellular ligands, their main role is related to metabolism of cholesterol associated with ApoE-containing lipoproteins, which influences the development of AD neuropathology (reviewed in Spuch et al., 2012). Moreover, LRPs can interact with APP, thereby promoting Aβ formation, and also participate in endocytic and internalization processing of ApoE, APP, and Aβ. Overall, LRPs have an important contribution to amyloid metabolism and accumulation which is worthy to explore. Other receptors known to bind Aβ oligomers are found at the postsynaptic membrane of excitatory synapses, and could be responsible for their neurotoxic effects. Among the Aβ potential targets are glutamate receptors, neurotrophin receptors, neuroligin, and other receptors that have been summarized in Dinamarca et al. (2012) review. In particular, further characterization of Aβ binding to neuroligin in a domain homologous to acetylcholinesterase may importantly contribute to understanding AD pathological mechanisms.

Related to PD, a dementia characterized by loss of cognitive skills and motor impairment, a main pathological hallmark is the aberrant aggregation of cytosolic α-synuclein (α-Syn) that culminates in neuronal death. However, emerging data have found extracellular aggregates of α-Syn, evoking a similar toxic pattern than Aβ, although their pathological actions are still unknown. One of the interesting purposed hypotheses exposed by Pacheco et al. (2012) is that extracellular α-Syn oligomers may form pores/perforations at the neuronal membrane which may provoke membrane disruption, thereby inducing neuronal impairments. Pacheco et al.'s work also provides a nice description of different pathways for unconventional exocytosis of soluble and aggregated α-Syn. These new insights will be worth to explore in order to unravel the pathological events leading to PD.

Last, but not least, related to brain friends, the work by Fiocchetti et al. (2012) brings us some hints on the neuroprotective actions of estradiol involving estrogen receptor beta (ERβ actions at the plasma membrane). Rapid signaling through this receptor has been poorly characterized so far, but novel findings indicate that ERβ-related mechanisms are required for hippocampal synaptic plasticity. Moreover, part of ERβ signal transduction involves neuroglobin activities, which displays a brain protective function. Further work is required to characterize this novel mechanism of neuroprotection related to learning and memory preservation.

We hope this Issue will be a source of inspiration for FMPB readers.
